# Where are all the men? A review of the barriers and facilitators to participation in health promotion interventions

**DOI:** 10.1093/geroni/igab046.3069

**Published:** 2021-12-17

**Authors:** Britteny Howell, Sage Corbett, Jennifer Peterson

**Affiliations:** 1 University of Alaska Anchorage, Anchorage, Alaska, United States; 2 University of Alaska Fairbanks, Fairbanks, Alaska, United States

## Abstract

Research shows that men in the U.S. experience significant morbidity and earlier mortality than women and are less likely to access, interpret, and apply health information to improve their outcomes. Although evidence-based health promotion programs have proven successful at increasing healthy lifestyle behaviors and reducing morbidity among older adults, older males are still significantly less likely to enroll and sustain participation in such health interventions. While studies have shown the barriers and facilitators to older adult participation in health programs in general, it is largely unknown why older male recruitment and participation in health promotion interventions remains so low. In this poster presentation, we conducted a thorough review of the last 20 years of existing research across a variety of academic search databases to outline the barriers, facilitators, and recommendations for increasing older male participation in health promotion programs. Of 1,194 initial search results, 383 article abstracts were thoroughly screened for inclusion, and 26 articles met all inclusion criteria. Included studies were coded and analyzed using Grounded Theory and reveal that masculine gender roles, as well as program scope, environment, and gender of the instructors and other participants, were important factors for male participation. Interventions should include men in all aspects of program planning and implementation, take into account men’s existing relationships and interests to create gender-sensitive programming, and clearly delineate the benefits to participation. Lastly, the field of public health would benefit by helping to normalize men’s participation in health promotion interventions.

